# Exosomes Derived from Umbilical Cord Mesenchymal Stem Cells Alleviate Mifepristone-Induced Human Endometrial Stromal Cell Injury

**DOI:** 10.1155/2020/6091269

**Published:** 2020-01-25

**Authors:** Jianye Wang, Ruomeng Hu, Qiong Xing, Xinghao Feng, Xiaohua Jiang, Yuping Xu, Zhaolian Wei

**Affiliations:** ^1^Reproductive Medicine Center, Department of Obstetrics and Gynecology, The First Affiliated Hospital of Anhui Medical University, No. 218 Jixi Road, Hefei, 230022 Anhui, China; ^2^Anhui Province Key Laboratory of Reproductive Health and Genetics, Anhui Medical University, No. 81 Meishan Road, Hefei, 230032 Anhui, China; ^3^NHC Key Laboratory of Study on Abnormal Gametes and Reproductive Tract (Anhui Medical University), No 81 Meishan Road, Hefei, 230032 Anhui, China

## Abstract

The human endometrial stromal cells (hEndoSCs) could maintain endometrial homeostasis and play a critical role in repairing endometrial injury. Mesenchymal stem cells (MSCs) significantly increase the proliferation of damaged hEndoSCs and protect them from apoptosis. Recent studies indicated that exosomes derived from stem cells could be recruited to damaged tissues for regeneration, which exhibit the potential for stem cell therapy as therapeutic vectors. In this study, we isolated human umbilical cord mesenchymal stem cell-derived exosomes (hUCMSC-Exos) and investigated the effects of hUCMSC-Exos on mifepristone-induced hEndoSC injury. Exosome uptake and cell proliferation as well as cell apoptosis of damaged hEndoSCs treated with hUCMSC-Exos were detected. We also assessed the expression of apoptosis-related proteins and the PTEN/AKT signaling pathway. We found hUCMSC-Exos improved the proliferation of damaged hEndoSCs and protected hEndoSCs from the mifepristone-induced apoptosis. hUCMSC-Exos upregulated Bcl-2 level as well as downregulated Cleaved Caspase-3 level and activated the PTEN/AKT signaling pathway to regulate the proliferation and antiapoptosis. These results indicated hUCMSC-Exos protected hEndoSCs from mifepristone-induced apoptosis and played an active role in repairing the damaged hEndoSCs through the PTEN/AKT signaling pathway *in vitro*. hUCMSC-Exos may hold great promise in the cell-free therapy of endometrial injury.

## 1. Background

The endometrium is a highly regenerative tissue, which undergoes monthly growth, differentiation, and shedding during a woman's reproductive years. Human endometrial stromal cells play an essential role in maintaining endometrial homeostasis [1]. Studies supported that hEndoSCs were responsible for endometrial decidualization, vascular reconstruction, immune cell recruitment, and plentiful molecule production, which play a critical role in repairing endometrial injury [2]. Endometrial injury may lead to hEndoSC apoptosis and the decrease in hEndoSCs viability, which induced endometrial atrophy and failure of endometrial homeostasis [3]. Endometrial injury activates apoptotic signaling pathways, inhibits endometrial angiogenesis, and hinders endometrial regeneration.

Mesenchymal stem cells (MSCs) have self-renewal and multipotential differentiation properties and are widely applied into the stem cell therapy [4]. MSCs derived from umbilical cord, one of the abundant sources of MSCs, provide a safe and low immunogenic alternative source of tissue regenerating after endometrial injury [5, 6]. Stem cell therapy opens up new perspectives for the treatment of endometrial injury. MSCs have been transplanted in the mouse model of Asherman's syndrome, and the result revealed that MSCs could improve the fertility of an injured endometrium [7, 8]. After the damaged hEndoSC coculturing with MSCs in vitro, MSCs significantly increased the rate of proliferation and decreased apoptosis [9]. And many patients had already been pregnant after the transplantation of MSCs with collagen scaffolds [10, 11].

Exosomes, membrane vesicles (30-150 nm in diameter), act as intracellular cargos carrying proteins, lipids, and nucleic acids as well as cell surface markers [12]. Recent studies indicated that exosomes derived from stem cells functioning as paracrine factors could regenerate cells and tissues and exhibit the potential for stem cell therapy as therapeutic vectors [13]. The exosomes secreted by stem cells not only contain the specific pluripotent transcriptional factors but also contain abundant noncoding RNAs to regulate the expression of related genes in receptor cells and promote the repair and regeneration of receptor cells [14, 15]. The exosomes, as mediators of intercellular communication, can upregulate the antiapoptotic genes and downregulate the proapoptotic genes, thus inhibiting the apoptosis of damaged cells or tissue and promoting tissue repair and regeneration [16, 17]. Many studies demonstrated hUCMSC-Exos could coordinate the immune system and regulate inflammatory responses in damaged tissues, which excite new avenues for cell-free therapy of many diseases [18]. However, mechanisms coordinating stem cell therapy of endometrial injury remain unclear. In this study, we research the recovery potential of exosomes derived from hUCMSCs for the treatment of damaged human endometrial stromal cells and mechanisms of hUCMSC-Exos on repair endometrial injury *in vitro*.

## 2. Materials and Methods

### 2.1. Isolation and Characterization of hUCMSCs

hUCMSCs were isolated from the connective tissue of the human umbilical cord as described [19]. After enzymatic digestion, hUCMSCs was cultured in expansion medium consisting of Dulbecco's modified Eagle's medium (DMEM)/F-12 supplemented with 10% (*v*/*v*) fetal bovine serum (FBS, Gibco, USA). The typical markers of hUCMSCs were detected by a flow cytometer (BD FACSCalibur, USA), such as specific antibodies for CD34 (1 : 1000, Abcam, UK), CD45 (1 : 1000, Abcam, UK), CD73 (1 : 1000, Abcam, UK), CD90 (1 : 1000, Abcam, UK), CD105 (1 : 1000, Abcam, UK), and HLA-DR (1 : 1000, Abcam, UK) conjugated with FITC and APC, respectively. The multilineage differentiation potential (adipogenic, osteogenic, and chondrogenic differentiation) of hUCM-MSCs was examined to check the stemness.

### 2.2. Isolation and Characterization of hUCMSC-Derived Exosomes

hUCMSC-Exos isolated from culturing medium with exosome-free FBS using ExoQuick-TC (System Biosciences, USA). Hence, hUCMSC-Exos were prepared for the following experiments: Exosomes were characterized by negative-staining electron microscopy and western blotting according to their size and surface marker expression. Exosomes were loaded into a formvar/carbon-coated grid, negatively stained with 3% aqueous phosphor-tungstic acid for 1 min, and observed by transmission electron microscopy (TEM, FEI, USA) at an accelerating voltage of 120 kV. The typical markers of exosomes were identified by western blotting, such as CD63 (1 : 1000, Abcam, UK), PDCD6IP (1 : 1000, Abcam, UK), TSG101 (1 : 1000, Abcam, UK), and LC3A (1 : 1000, Abcam, UK). Further, particle size distribution for the hUCMSC-Exos was measured by High-Sensitivity Flow Cytometry (NanoFCM, UK).

### 2.3. Isolation and Characterization of hEndoSCs

Endometrial tissues were obtained from patients with a normal endometrium in the proliferative phase. We slightly scratched the endometrium of patients to improve their success of embryo transfer. All patients were healthy fertile women who had regular menstrual cycles of 25-30 days and had not used hormonal contraception, intrauterine device, or received hormone therapy for at least 3 months before surgery. Endometrial cycle and endometrial thickness (i.e., the architecture of the lining and endometrial stripe) and pathology were determined according to the patient's menstrual history, B-sonography, and histologic examination.

Fresh endometrial specimens in a proliferative phase were rinsed, minced into small pieces (1-2 mm^3^), and then incubated in 0.1% type I collagenase (Sigma, USA). The dispersed endometrial cells were separated by filtration through successive nylon sieves (180 *μ*m and 40 *μ*m nylon sieve). hEndoSCs were maintained in DMEM/F-12 with 10% FBS (Gibco, USA) and 1% penicillin/streptomycin (Hyclone, USA) [20].

To detect typical markers of hEndoSCs, the cells were identified by specific antibodies for CD34 (1 : 1000, Abcam, UK), CD44 (1 : 1000, Abcam, UK), and CD90 (1 : 1000, Abcam, UK) conjugated with PE/Cy5.5, FITC and PE, respectively. After incubation at room temperature, the cells were examined with a flow cytometer (BD FACSCalibur, USA).

### 2.4. Establishment of the Human Endometrial Stromal Cell Injury Model

ESCs at passages 3-5 were harvested, dispensed into well culture plates, and cultured in DMEM/F-12 with10% FBS for 48 hours. This medium was changed to a medium without FBS, and the cells were treated with 60 *μ*mol/L mifepristone (Sigma, USA) for 48 hours. After the treatment, the medium was changed with a fresh medium to continue culturing and mifepristone was withdrawn. The damaged endometrial stromal cell model was established *in vitro*.

### 2.5. Quantitative Uptake Efficiency of hUCMSC-Exos

PKH26 (Life Technologies, USA) was used to label hUCMSC-Exos. hEndoSCs were cultured in 96-well plates with mifepristone for 48 h. After mifepristone administration, PHK26-labelled hUCMSC-Exos were resuspended in serum-free medium and cultured with endometrium stromal cells of each well. After culturing the cells for 0.5 h, 1 h, 2 h, 4 h, 8 h, 14 h, and 20 h, we observed the cells by confocal scanning laser microscopy (LSM800, Zeiss, Germany) and detected fluorescence intensity of PKH26.

### 2.6. Proliferation Assay by Cell Counting Kit 8 Assay (CCK8)

Cell proliferation was determined by CCK-8 kit (Dojindo, Japan) according to the manufacturer's instructions. Briefly, hEndoSCs were seeded in 96-well plates with medium containing 10% FBS and incubated for 24 h in a humidified incubator at 37°C for adhesion; then we replaced the medium with a serum-free medium added with 60 *μ*mol/L mifepristone and cultured for 48 h. The medium was replaced with DMEM/F-12 added with hUCMSC-Exos using DMEM/F-12 as the control. Then, the absorbance at 450 nm was measured by a microplate reader.

### 2.7. Flow Cytometric Analysis of Cell Apoptosis

hEndoSCs in 6-well culture plates were harvested and terminated by aspirating the cultivating supernatant fluid, and cells were washed twice in PBS. Cells were then incubated with FITC-conjugated Annexin V and propidium iodide (Vazyme Biotech Co., Ltd., China) in binding buffer for 15 minutes at room temperature without exposure to light and analyzed by flow cytometry (BD FACSCalibur, USA). This test discriminates 4-cell populations, intact cells (Annexin V^−^/PI^−^), early apoptotic cells (Annexin V^+^/PI^−^), necrotic cells (Annexin V^−^/PI^+^), and late-apoptotic/necrotic cells (Annexin V^+^/PI^+^). Ten thousand cells were routinely acquired, and the results were expressed as the percentage of apoptotic Annexin V^+^ and necrotic PI^+^ cells of total cells counted. The results were analyzed with FlowJo software.

### 2.8. Intracellular Signaling Evaluation by Western Blot Analysis

After appropriate periods of cultivation, cells were washed twice with PBS and scraped into lysate buffer containing 1 mM dithiothreitol (DTT), 1 mM phenylmethylsulfonyl fluoride (PMSF), 1 mg/mL leupeptin, 2 mg/mL aprotinin, and 5 mM EGTA in PBS. The cells were sonicated with a sonifier cell disrupter, and sonicates were then centrifuged at 10000 × g for 10 min. The supernatants were denatured in sample buffer and heated in boiling water for 5 min. Proteins were separated by 10% SDS-PAGE and transferred electrophoretically from the gels to polyvinylidene difluoride (PVDF) transfer membranes. The membranes were incubated for 2 h in a blocking solution containing 5% skim milk and 0.05% Tween-20 in PBS (PBS-Tween). The membranes were washed briefly in PBS-Tween and incubated with the Bcl-2 (1 : 1000, Abcam, UK), Caspase-3 (1 : 1000, Abcam, UK), Cleaved Caspase-3 (1 : 1000, Cell Signaling, USA), PTEN (1 : 1000, Cell Signaling, USA), AKT (1 : 1000,Cell Signaling, USA), and p-AKT (1 : 1000, Cell Signaling, USA) antibody at 4°C overnight. The membranes were next washed 3 times in PBS-Tween by using a rotary shaker. The washed membranes were incubated with horseradish peroxidase- (HRP-) conjugated anti-rabbit for 1 h. The membranes were washed again and processed with an ECL detection kit (Biosharp, USA) according to the manufacturer's direction to visualize the proteins recognized by the antibodies.

### 2.9. Statistical Analysis

All data are presented as the mean ± standard deviation from at least three independent runs. All statistical analyses were performed using Student's two-tail paired *t*-test. A *p* value < 0.05 was considered statistically significant. An asterisk (^∗^) indicates *p* < 0.05, and two asterisks (^∗∗^) indicate *p* < 0.01.

## 3. Results

### 3.1. Characterization of hUCMSCs and hUCMSC-Exos

**Figure 1:**
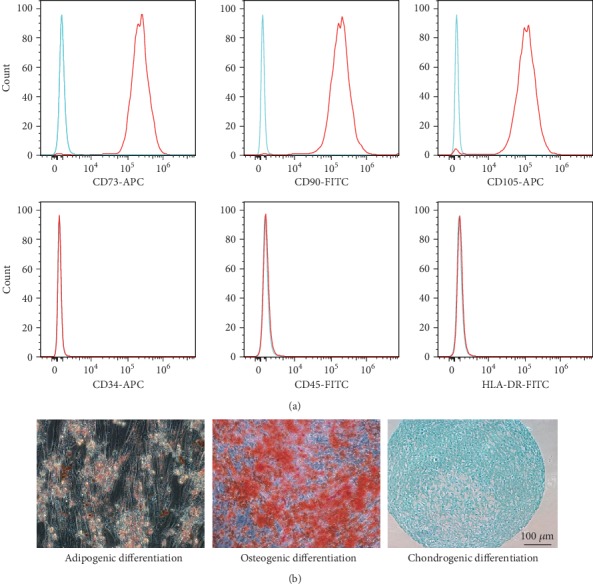
Typical characteristics of hUCMSCs. (a) The positive markers (CD73, CD90, and CD105) and negative markers (CD34, CD45, and HLA-DR) of hUCMSCs were detected by a flow cytometer. (b) The multilineage differentiation potential (adipogenic, osteogenic, and chondrogenic differentiation) of hUCM-MSCs were checked.

To identify the hUCMSCs, the positive markers for mesenchymal stem cell markers (CD73, CD90, and CD105) and the negative markers (CD34, CD45, and HLA-DR) were checked by fluorescence-activated cell sorting (FACS). The results demonstrated that the positive markers, including CD73, CD90, and CD105, were highly expressed. Furthermore, the negative markers (CD34, CD45, and HLA-DR) were not expressed (Figure 1(a)). The differentiation potential of hUCMSCs into adipocytes, osteoblasts, and chondroblasts was confirmed (Figure 1(b)).

**Figure 2:**
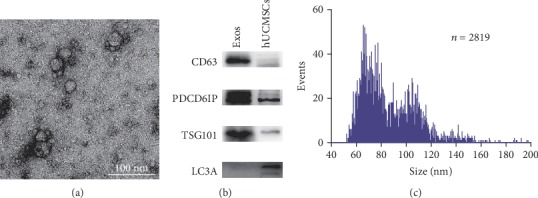
Typical characteristics of hUCMSC-Exos. (a) Morphology of hUCMSC-Exos obtained by transmission electron microscopy. (b) The expression levels of CD63, PDCD6IP, HSPA8, and TSG101 in hUCMSC-Exos were detected by western blotting. (c) Histogram of particle size distribution for the hUCMSC-Exos sample by flow nanoanalyzer analysis (*n* = 2819); the average diameter of hUCMSC-Exos was 86.79 ± 22.70 nm.

To characterize the hUCMSC-Exos, we extracted exosomes from the hUCMSC culture medium using the ExoQuick-TC reagent. hUCMSC-Exos were light yellow substance precipitated in the bottom of the tube. The exosome morphology and size were visualized by TEM, and the diameters of isolated exosomes were 30-100 nm as spherical shapes (Figure 2(a)). Western blot confirmed that hUCMSC-Exos expressed exosome-specific markers CD63, PDCD6IP, and TSG101, but not the negative marker autophagosome protein LC3A (Figure 2(b)). Flow nanoanalyzer analysis showed that the average diameter is 86.79 ± 22.70 nm (Figure 2(c)), and the concentration of the hUCMSC-Exos was approximately 3.17 × 10^11^ particles/mL. These data demonstrate that hUCMSC-Exos were successfully extracted and purified from hUCMSC culture medium.

### 3.2. Characterization of hEndoSCs

**Figure 3:**
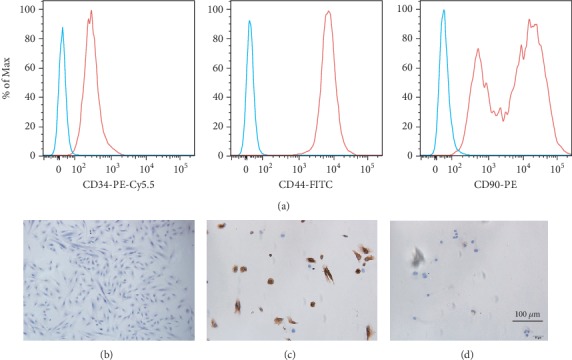
Typical characteristics of hEndoSCs. (a) Flow cytometry detection of the immunophenotypes of hEndoSCs. The cells were positive for CD34, CD44, and CD90. (b) HE staining showed morphology of hEndoSCs. (c) Immunohistochemical identification of vimentin expression. (d) Immunohistochemical identification of keratin expression.

The hEndoSCs were isolated from fresh endometrial specimens in a proliferative phase. The immunophenotypes of hEndoSCs were analyzed by a flow cytometer, and the analysis results showed the cells were positive for CD34, CD44, and CD90 (Figure 3(a)).  Figure 3(b) shows the morphology of hEndoSCs. At the same time, the cells were assessed for their expression of vimentin and keratin, and the immunohistochemical identification showed that the vimentin and keratin were expressed in cells (Figures 3(c) and 3(d)).

### 3.3. Effective Uptake of hUCMSC-Exos by Mifepristone-Injured hEndoSCs

**Figure 4:**
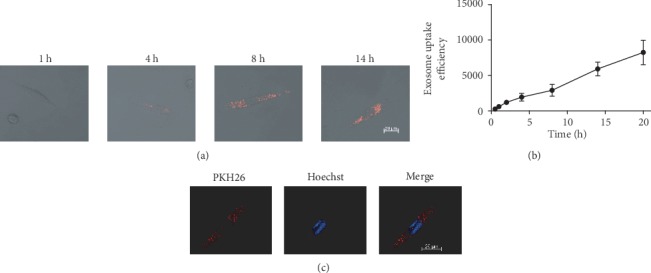
PHK26-labelled hUCMSC-Exos were uptook by injured hEndoSCs and quantitative uptake ratio of hUCMSC-Exos. (a) Confocal microscopy detection of PKH26-labelled hUCMSC-Exos uptake by hEndoSCs at different time points. (b) The uptake of hUCMSC-Exos by hEndoSCs is time-dependent. (c) hUCMSC-Exos are adsorbed and engulfed in injured hEndoSCs.

To verify hUCMSC-Exos uptake by damaged hEndoSCs, we labelled hUCMSC-Exos with PKH26 and then cocultured PHK26-labelled hUCMSC-Exos with mifepristone-induced hEndoSCs. The confocal scanning laser microscopy was used to monitor the efficiency of exosome uptake by damaged hEndoSCs in real time.  Figure 4(a) shows the typical PKH26-labelled positive cells at different time points (1 h, 4 h, 8 h, and 14 h) after hUCMSC-Exos taken up by damaged hEndoSCs.  Figure 4(b) shows the statistical results from the different time points (0.5 h, 1 h, 2 h, 4 h, 8 h, 14 h, and 20 h). The exosome uptake efficiency increased with the number of hUCMSC-Exos (red fluorescence) adsorbed or engulfed by the hEndoSCs. The hEndoSCs were labelled with Hoechst, and we found hUCMSC-Exos gathered in the interior of the cells around the cell nucleus (Figure 4(c)).

### 3.4. hUCMSC-Exos Promote the Proliferation of Damaged hEndoSCs

**Figure 5:**
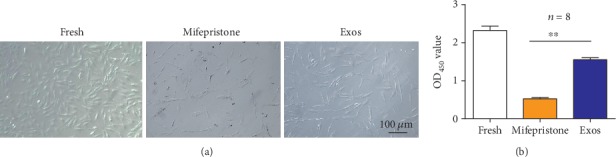
hUCMSC-Exos promote the proliferation of damaged hEndoSCs. (a) Morphology of hEndoSCs in different groups. (b) Proliferation of the mifepristone-induced hEndoSCs after coculturing with hUCMSC-Exos. The proliferation of the Exos groups was larger than that of the mifepristone group (^∗∗^*p* < 0.01).

The hEndoSCs were divided into three groups: hEndoSCs cultured in DMEM/F-12 with 10% FBS (fresh group), mifepristone-induced hEndoSCs in serum-free medium (mifepristone group), and mifepristone-induced hEndoSCs cocultured with hUCMSC-Exos (exosome group).  Figure 5(a) shows the fresh hEndoSCs were cultured at ~70% confluent. After being treated with mifepristone for 48 h, a large population of hEndoSCs was dead and the confluency of damaged hEndoSCs was ~30%. Otherwise, the damaged hEndoSCs recovered after co-culturing with hUCMSC-Exos and the confluent of the exosome group was ~50%.

To determine the effect of hUCMSC-Exos on damaged hEndoSCs, we obtained proliferation of damaged hEndoSCs using the CCK-8 kit after hEndoSCs treated with hUCMSC-Exos for 48 h.  Figure 5(b) shows the proliferation of three groups, and the proliferation of the exosome group was significantly higher than that of the mifepristone group. The analysis results showed that there was a significant difference between the Exos group and mifepristone group. Our data indicated hUCMSC-Exos could promote the proliferation of damaged hEndoSCs.

### 3.5. hUCMSC-Exos Protect hEndoSCs from Mifepristone-Induced Apoptosis *In Vitro*

**Figure 6:**
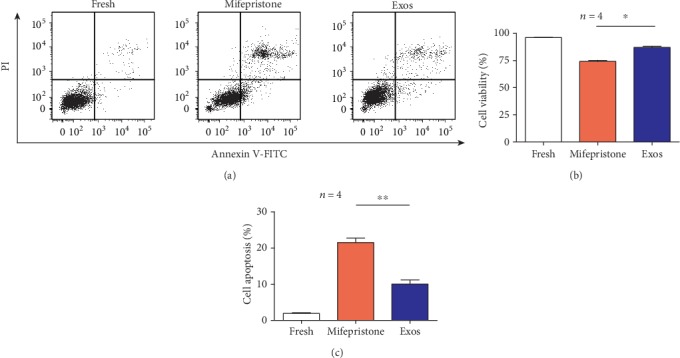
hUCMSC-Exos protect against mifepristone-induced injury of hEndoSCs and promote resistance to cell apoptosis *in vitro*. (a) Flow cytometry detection of hEndoSCs apoptosis in the fresh, mifepristone, and Exos groups. (b) Through Annexin V-FITC/PI double staining and FACS analysis, there was a statistically significant difference in the proportion of living cells between the mifepristone group and Exos group (*p* < 0.05). (c) The percentage of apoptotic cells; a significant difference was observed between the mifepristone group and Exos group (*p* < 0.01).

To further investigate the effect of hUCMSC-Exos on damaged hEndoSCs, we assessed the cell apoptosis by a flow cytometer. As determined by Annexin V-FITC/PI staining and FACS analysis, the percentages of living cells and apoptotic cells in the fresh group were 96.5% and 2.1%. The living cell rate in the mifepristone group was 73.8% and the apoptotic cell rate was 21.9%, and the corresponding rates in the Exos group were 87.0% and 8.71% (Figure 6(a)). The mifepristone obviously induced the hEndoSCs apoptotic, and the proportion of living cells significantly reduced after mifepristone administration.  Figure 6(b) shows the percentages of living cells in the Exos group compared with those in the mifepristone group were significantly different (*p* < 0.05). Meanwhile, there were significant differences in the percentage of apoptotic cells between the mifepristone group and Exos group (*s*) as shown in Figure 6(c). These results showed that the apoptosis rate of hEndoSCs was significantly increased after mifepristone treatment. And hUCMSC-Exos could alleviate mifepristone-induced apoptosis of hEndoSCs.

### 3.6. hUCMSC-Exos Alleviate the Apoptosis through Activation AKT Signaling Pathways in Damaged hEndoSCs

**Figure 7:**
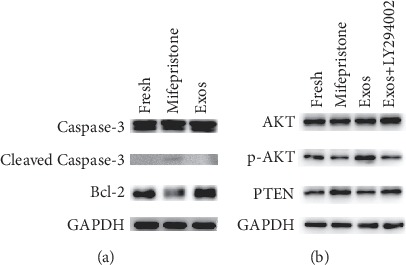
hUCMSC-Exos regulate hEndoSC apoptosis and activate the PTEN/AKT signaling pathway *in vitro*. (a) The expression levels of Bcl-2, Caspase-3, and Cleaved Caspase-3 were detected by western blotting in the fresh, mifepristone, and Exos groups. (b) The expression levels of p-AKT, AKT, and PTEN were detected by western blotting in the fresh, mifepristone, Exos groups and Exos groups treated with a PTEN/AKT pathway inhibitor (LY294002).

To further investigate the effect of hUCMSC-Exos, western blotting was used to detect the expression of Bcl-2, Caspase-3, and Cleaved Caspase-3 (Figure 7(a)). The positive expression of Cleaved Caspase-3 increased after mifepristone administration, but the expression of Caspase-3 was not affected. On the contrary, the Bcl-2 expression was found to be significantly decreased. However, the expression of Caspase-3 and Cleaved Caspase-3 in the Exos group was reduced after hUCMSC-Exos was added, and the Bcl-2 expression was increased. These findings reveal that hUCMSC-Exos enhance antiapoptosis and have a robust protective effect on the mifepristone-induced damage of hEndoSCs.

The PTEN/AKT signaling pathway plays an important role in cell apoptosis and angiogenesis. To further assess the molecular mechanism of protection of hUCMSC-Exos, we assessed the level of PTEN, AKT, and p-AKT in four groups (Figure 7(b)). In the Exos group, the level of p-AKT expression significantly increased. However, this increase was not associated with an increase in total AKT expression, and there was no change in the total AKT protein level. In the Exos group treated with a PTEN/AKT pathway inhibitor (LY294002), the expression of p-AKT decreased and remained higher compared with that of the mifepristone group. A negative correlation between PTEN and p-AKT expression was observed. These results demonstrated that hUCMSC-Exos could induce the overexpression of p-AKT and activate the PTEN/AKT signaling pathway which could regulate cell growth, migration, and angiogenesis, and this effect was partly weakened by the PTEN/AKT pathway inhibitor.

## 4. Discussion

Studies have previously demonstrated that hUCMSCs repaired the injured tissue and protected against apoptosis [9, 21]. However, the exact mechanisms were unclear. In this study, we extracted exosomes derived from hUCMSCs and cocultured with the mifepristone-induced endometrial stromal cells. We found that hUCMSC-Exos could improve the survival of damaged hEndoSCs and alleviate the apoptosis of hEndoSCs induced by mifepristone. We determined hUCMSC-Exos could activate the PTEN/AKT signaling pathway in mifepristone-injured hEndoSCs, and further upregulated Bcl-2 as well as downregulated Cleaved Caspase-3. After all, our results indicated that hUCMSC-Exos played an active role in repairing the injured endometrium through the PTEN/AKT signaling pathway.

Compared with normal endometrium, the molecular, morphological, and functional characterization of damaged endometrium entirely changed, and damaged endometrial cells lead to apoptosis. Mifepristone inhibited the Bcl-2 expression and induced endometrial cell apoptosis [9]. After mifepristone administration, damaged hEndoSCs had been established by the molecular, morphological, and functional characterization. We obtained the cell growth was inhibited, and apoptosis was induced by mifepristone in vitro. Western blotting showed that expression of Cleaved Caspase-3 increased and expression of Bcl-2 decreased in mifepristone-induced hEndoSCs. And our findings indicated that mifepristone could promote endometrial apoptosis by upregulation of PTEN expression. The PTEN/AKT signaling pathway could regulate proliferation, differentiation, and migration of endometrial cells. Previous studies showed that the activation of the PTEN/AKT signaling pathway upregulates VEGF and VEGFR-2 expression to induce the repair of an injured endometrium [22]. MSCs could improve the recovery of damaged endometrial cells from the stress and apoptosis induced by mifepristone *in vitro* and inhibited apoptosis by downregulation of Caspase-3, Caspase-8, and Caspase-9 in hEndoSCs [9].

We isolated hUCMSC exosomes and confirmed the characteristics. Western blotting analyses the characteristic protein expression of exosomes such as CD63, PDCD6IP, TSG101, and LC3A. Moreover, it was observed that hUCMSC-Exos could be absorbed by injured hEndoSCs *in vitro*. We cocultured damaged endometrial cells with hUCMSC-Exos and checked the effects of hUCMSC-Exos on the damaged hEndoSCs. The expression of Bcl-2 obviously was upregulated, and the expression of Cleaved Caspase-3 was downregulated after hUCMSC-Exos administration. The level of p-AKT expression significantly increased, and a negative correlation between PTEN and p-AKT expression was observed. We proposed that hUCMSC-Exos protected the hEndoSCs from mifepristone-induced apoptosis through the PTEN/AKT signaling pathway and activated the AKT to regulate of Bcl-2 and Cleaved Caspase-3 expression.

Many studies have demonstrated that a variety of miRNAs were observed in hUCMSC-Exos [23, 24]. miRNAs are a series of small noncoding RNAs (~22 nucleotides long) that regulate the expression of target genes at the posttranscriptional level. During this process, the miRNA/miRNA-induced silencing complex (miRISC) binds the 3′-UTR of target mRNA to inhibit expression via translational repression and/or mRNA degradation [25]. It was found that miRNAs regulate the phosphorylation of AKT through PTEN expression in various tissues and many antiapoptotic factors such as Bcl-xL inhibit the activation of the apoptotic pathway and increase the antioxidant activity of cells [26, 27]. Exosomes contain abundant miRNAs to regulate the expression of related genes in receptor cells and promote the regeneration and repair of receptor cells. It has been reported that exosomal miR-32-5p induced multidrug resistance by inhibited PTEN expression and activates the PI3K/AKT pathway [28] and miR-21-5p dysregulation in exosomes augmented AKT kinase activity and impaired the regenerative activities [29]. We will further research the mechanism of microRNAs transported by exosomes derived from hUCMSCs in the repair of endometrial injury and practice the exosomes in vivo and in clinical therapy for endometrium injury.

In summary, we found hUCMSC-Exos could promote cell survival and proliferation of damaged hEndoSCs and alleviate hEndoSCs from the mifepristone-induced apoptosis. hUCMSC-Exos activated the PTEN/AKT signaling pathway and regulated the hEndoSCs apoptosis. The results of the present study lay the foundation for further studies on the potential application of hUCMSC-Exos in human endometrial injury.

## Data Availability

The data that support the findings of this study are available upon request from the corresponding author. The data are not publicly available due to privacy concerns or ethical reasons.
